# Confusion and a cough: an experience of COVID-19 in dementia patients

**DOI:** 10.1192/bjo.2021.121

**Published:** 2021-06-18

**Authors:** Emily Giles

**Affiliations:** NHS Lanarkshire

## Abstract

**Aims:**

To assess the clinical presentation and outcomes of COVID-19 positive patients with dementia and to evaluate the suitability of the “4C mortality score.” Older adults with dementia are a vulnerable patient group therefore it was predicted that this patient demographic would have poorer outcomes and high mortality rates. Ward 24 is an organic old age psychiatry ward in University Hospital Monklands, Lanarkshire for patients with advanced dementia. Older adults have been found to have atypical presentations and non-specific symptoms in COVID-19, however given COVID is still a new and evolving disease, little is known about the impact on dementia patients. The 4C mortality score was designed to predict in-hospital mortality for hospitalised COVID-19 patients using a number of clinical parameters.

**Method:**

Data were collected retrospectively from all inpatients on ward 24 testing positive for COVID-19 between October and December 2020. Data were collected using online MIDIS entries, paper notes, NEWS charts and Clinical Portal. A 4C mortality score was calculated for each patient using an online calculator based on the data collected.

**Result:**

15 patients tested positive for COVID-19; 47% male and 53% female, age range between 64 and 92 years old. 67% of patients had 3 or more comorbidities and 89% had either a high or very high 4C mortality score. Mortality from COVID-19 was 13% and 20% of patients required oxygen. 27% of patients were asymptomatic, these patients also had the lower risk mortality scores. 67% presented with pyrexia, 33% had a cough and 13% had breathlessness. Non-specific symptoms were also seen; 53% had fatigue, 20% had diarrhoea and 20% had unresponsive episodes. Post COVID delirium was seen in 20% of patients.

**Conclusion:**

Mortality rates were lower than expected, indicating that the 4C mortality score might not be appropriate to use in this patient demographic due to confounding factors. Atypical symptoms were common in patients, with a variability of clinical presentations within the patient demographic. These findings suggest the importance of having a low threshold for COVID-19 infection even in the absence of typical symptoms. Development of an alternative risk stratification tool would be beneficial for this patient group, with further studies needed on a larger scale to facilitate this.

